# Risk factors for depression in elderly diabetic patients and the effect of metformin on the condition

**DOI:** 10.1186/s12889-019-7392-y

**Published:** 2019-08-07

**Authors:** Fenqin Chen, Guozhu Wei, Yingfang Wang, Tingting Liu, Ting Huang, Qian Wei, Guojing Ma, Difei Wang

**Affiliations:** 1 Department of Geriatrics, the First Affiliated Hospital, China Medical University, Shenyang, 110001 China; 2 Department of Radiology, Orthopedic Hospital of Shenyang, Shenyang, 110001 China

**Keywords:** Metformin, Depression, Elderly

## Abstract

**Background:**

At present, only a few studies have focused on the risk factors for depression in elderly diabetic patients, and there is little evidence for the effect of metformin in depressed elderly patients with diabetes than on its effect on blood glucose. The aim of the current work was to study the risk factors for depression in elderly diabetic patients and to ascertain the effects of metformin on the depressive state.

**Methods:**

We initiated a 1:4 matched case–control study. The case group comprised 110 elderly diabetic patients with depression from nine communities in Shenyang in 2017. The control group comprised 440 non-depressed elderly diabetic patients from the same communities, which were matched by gender and age (± 2 years of age) with the case group. Depression was measured using the Geriatric Depression Scale-15, and we performed matched univariate and multivariate logistic regression analyses.

**Results:**

In the multivariate analysis, overweight status, poor physical capabilities and low activity level, and the presence of more than two additional illnesses were risk factors for depression in elderly patients with diabetes. For these risk factors, the adjusted ORs (all *P* < 0.05) were as follows: an adjusted OR of 2.031 and 95% CI of 1.180–3.495; an adjusted OR of 2.342 and 95% CI of 1.465–3.743; and an adjusted OR of 5.350 and 95% CI of 2.222–12.883, respectively. Patients taking metformin had a lower risk of depression than those taking no medication, with an adjusted OR of 0.567 and 95% CI of 0.323–0.997 (*P* < 0.05).

**Conclusions:**

Overweight status, poor physical capabilities and low activity level, and the presence of more than two additional illnesses were risk factors for depression in elderly diabetic patients, and metformin was a protective factor against depression in elderly diabetic patients.

## Background

China has a large, rapidly expanding elderly population, and it currently leads the world in this category. Since 1980, China’s population of individuals over 60 years of age has, in fact, grown by an average of 3.2% annually; and, currently, the proportion of those individuals 60 years and older in China is 17.3% of the total. In 2017, China became the only country in the world to have more than 200 million elderly [1].

Diabetes mellitus, a chronic metabolic disease, has reached epidemic status; and it now poses one of the major threats to human health of the twenty-first century. In 2017, the International Diabetes Federation (IDF) estimated that 425 million individuals worldwide were suffering from diabetes mellitus, and it is expected that the number will rise to 629 million in 2045 [2]. Complications of diabetes mellitus can cause increased morbidity, disability, and mortality, enough to pose a threat to the economies of all countries, especially those that are still developing [3]. Over the past few decades, prediabetes and diabetes have become more common in both the developed and developing parts of China. A recent national study of a representative sample of 170,287 adults from 31 provinces on the Chinese mainland reported the age-standardized prevalence of diabetes and prediabetes to be 10.9 and 35.7%, respectively. The prevalence of elderly patients with diabetes and prediabetes was 20.2 and 45.8%, respectively [4].

Depression is a multifaceted phenomenon that involves loss of satisfaction, hope, energy, and interest; and it is often accompanied by feelings of helplessness, worthlessness, boredom, and a loss of interest in previously enjoyed activities [5, 6]. The prevalence of depression also differs across different parts of China. Presently, diabetes and depression are prevalent in both developed and developing countries; and it has been confirmed that diabetes can increase the risk of depressive symptoms to some extent, while depression can increase the risk of developing diabetes [7, 8]. Depression can impinge on a patient’s self-management ability and hinder her or his adherence to treatment regimens [9]. Although there have been tremendous advancements in the diagnosis and treatment of diabetes, depression in diabetic patients remains underdiagnosed and undertreated [10]. Unrecognized and untreated depression in diabetic patients lead to a higher prevalence of depression and a probability of greater severity; which in turn causes poor glycemic control, lower adherence to medication, higher treatment costs, and a higher mortality rate [11]. Furthermore, the available studies have suggested that the presence of depression—co-morbid with diabetes—is associated with a poorer self-reported health status and more adverse outcomes [12–14]. According to other published studies, the prevalence of depression is approximately 20% of patients with type 2 diabetes mellitus (T2DM) [14, 15]. A meta-analysis by Knol found that adults with depression have a 37% increased risk of developing T2DM [16]. In another study, the authors observed a bidirectional relationship between new-onset diabetes and depression, and showed depression to be associated with a 60% increased risk of T2DM; whereas T2DM showed a more modest association with depression [17]. The great increase in the risk of co-morbid depression in diabetic patients might be attributed to the psychosocial burden of disease, poor social support, awareness of having a chronic disease or its related complications and disabilities, and the consequential psychologic burden [11]. Also, co-morbid depression among persons living with diabetes is associated with poor markers of diabetes control, including glycemic control, retinopathy, nephropathy, neuropathy, micro-vascular complications, and sexual dysfunction [18].

Metformin, which is widely used as a first-line treatment for patients with T2DM, has been in use for over 60 years. It can reduce hepatic glucose output and increase the insulin-mediated utilization of glucose [19, 20]. In addition to its hypoglycemic activity, metformin has been shown to elicit marked anti-inflammatory, antioxidant, and neuroprotective activities and to improve memory function and learning [10, 21, 22]. Another study also indicated that metformin treatment (especially long-term usage) was associated with a lower risk of cognitive impairment in older adults with diabetes [23]. The pleiotropic pharmacologic activities of metformin, then, makes it suitable for the treatment of diabetes mellitus and co-morbid depression, the latter involving a myriad of pathophysiologic characteristics. The aforementioned study confirmed that metformin improved hyperglycemia and depressive-like behavior primarily through synergistic effects on the HPA axis and on oxidative stress and monoamine transmission [10]. The Saghar study also suggested that metformin ameliorated methamphetamine-induced depression, cognition impairment, and neurodegeneration in rats via the CREB/BDNF and Akt/GSK3 signaling pathways [24].

Various studies on diabetes and depression and on depression and the elderly have been published [25, 26]. However, only a few studies have focused on the risk factors for depression in elderly diabetic patients, and there is less evidence for the effects of metformin in depressed elderly patients with diabetes than for its effect on blood glucose. Our primary aim, therefore, was to identify the risk factors for depression in elderly diabetic patients and to determine the effects of metformin on this group.

## Methods

### Study design and participants

We used United Nations (UN) guidelines, which define elderly individuals as 60 years old and older [27]. The subjects in our study were examined and recruited from May to October 2017, and the source population consisted of nine residential communities in Shenyang, Liaoning, located in northeastern China. We used the Geriatric Depression Scale (GDS) score and selected a case group of 110 elderly diabetic subjects with depression and a control group of elderly diabetic subjects without depression using 1:4 matching according to sex and age (+ 2 years). Our study was approved by the Ethics Committee of China Medical University (Shenyang, China, AF-SOP-07-1.0–01), and all procedures were conducted in accordance with ethical standards. All participants provided written consent after being informed of the use of their personal information, benefits of the study, inherent medical programs, and confidentiality agreements. For illiterate participants, we obtained written informed consent from their relatives.

### Study patient consent and measurements

Data were collected during a single visit to the clinic by endocrinologists and trained nurses in face-to-face interviews that used a standard questionnaire. Before the survey was administered, we required that all eligible investigators attend an organized training session. This training included providing the purpose of the study, instructions for administering the questionnaire, the standard methods of measurement used, the importance of standardization, and the study procedures themselves. All investigators were required to undergo rigorous testing after the training, and only those who scored perfectly on our test were allowed to proceed further. Our inspectors were given further instruction and support during the data collection period.

Data on demographic characteristics, such as name, gender, age, cultural level, marital status, health insurance coverage, pension status, previous medical history (including hypertension, cerebrovascular disease, diabetes, and other health problems), and lifestyle factors (such as diet, smoking, and alcohol consumption) were obtained by interview with a standardized questionnaire. Educational level was divided into primary school or less, middle school, and high school or more. Smoking status was divided into a non-smoking group, a smoking group, and a former smoking group. Drinking status over the past year was based upon consumption of alcohol beverage at least twice per week, with an average alcohol consumption of 40 g at one sitting; participants were divided into a non-drinking group, a drinking group, and a former drinking group. Physical capability and activity were divided into two groups: good ability and activity (can climb at least 4 flights of stairs) vs. poor ability and activity.

Physical examination included height and weight. We measured height and weight to the nearest 0.1 cm and 0.1 kg, respectively, while the subjects were fasting and wearing light-weight clothing, with their feet parallel and without shoes. Body mass index (BMI) was calculated as the weight in kilograms divided by the (height in meters) squared. The diagnostic criteria of obesity in China are derived from the common knowledge of experts on the prevention and treatment of adult obesity in China [28]. Individuals with BMI ≥24 kg/m^2^ were considered to be overweight and those with BMI ≥28 kg/m^2^ were considered to be obese.

Fasting blood samples from participants were collected by standard venipuncture, and separation of serum was performed by double centrifugation with a laboratory centrifuge. All blood samples were collected and stored at − 80 °C until analyzed. Plasma glucose levels were measured using the glucose oxidase method, and plasma glycated hemoglobin (HbA1c) levels were detected using an automated glycosylated hemoglobin analyzer (Bio-Rad, US).

The presence of type 2 diabetes mellitus for allocation into groups was diagnosed based upon the 1999 World Health Organization criteria [29]. A diagnosis of diabetes is often prompted by such symptoms as increased thirst and urine volume, recurrent infections, unexplained weight loss, and, in severe cases, drowsiness and coma. High levels of glycosuria are usually present, with a fasting plasma glucose concentration ≥ 7.0 mmol/L or 2 h post glucose load ≥11.1 mmol/L, or both. The diagnosis of diabetes should always be confirmed by repeating the test on another day unless there is unequivocal hyperglycemia with acute metabolic decompensation or obvious symptoms [29]. All health-related diseases except diabetes were self-reported, and this has shown satisfactory diagnostic accuracy in epidemiologic studies. In many cases, the findings have been further confirmed by medical records [30, 31].

We ascertained the depression status of patients at the time of recruitment using the Geriatric Depression Scale (GDS)-15 as the evaluation tool. The GDS-15 is a short, 15-item self-reporting scale for assessing depression and is a validated depression scale. Each item can have 2 answers, i.e., yes or no; the highest possible score is 15, which indicates the most severe depressive state. We also used a cut-off point of 5 or more, as the original version of GDS-15 had a sensitivity of 97% and a specificity of 95% [6, 32]. A score of 0–4 was considered normal; 5–8 indicated mild depression; 9–11 indicated the presence of moderate depression; and 12–15 indicated severe depression.

At baseline, anti-diabetic medications taken by the participants were ascertained from self-reports. Participants were divided into a no-medication group, metformin users, and those using other hypoglycemic drugs. Metformin users were further divided into subgroups. All metformin regimens in this group fell within a dosage range of 1.0–2.0 g/d, but some patients used metformin alone and others used it combined with other hypoglycemic drugs. Anti-diabetic medications (hypoglycemic agents) other than metformin included sulfonylureas, glinide class drugs, glycosidase inhibitors, thiazolidinediones, DPP-4 inhibitors, a GLP-1 receptor agonist, SGLT2 inhibitors, and insulin.

#### Exclusion criteria

In the first round of selection, we excluded participants who were non-diabetic, who were under 60 years of age, and those who had depression or other mental illnesses. We also excluded from analyses those individuals who had incomplete data, and those who had an illness that was either terminal (such as cancer), or who required urgent medical attention. The final participant population consisted of 550 subjects (110 in the case group and 440 matched controls) from a community in Shenyang City in Liaoning Province, China.

### Statistical analyses

EpiData was used to build the database. We performed conditional univariate and multivariate logistic regression analyses due to the matching design using SPSS 20.0 software. Odds ratios (OR) are reported with their respective 95% confidence intervals (CI), and values of *P* < 0.05 were considered statistically significant.

## Results

### General study conditions

A total of 550 participants were included in the present study: there were 110 elderly diabetic subjects with depression selected as the case group; and there were 86 cases who were classified as having mild depression, 14 cases with moderate depression, and 10 cases with severe depression. The mean age (± standard deviation) of the participants was 70.15 ± 6.50 years, with a male-to-female ratio of 1:1.6. There were an additional 440 elderly diabetic subjects without depression who were selected as the control group by 1:4 matching according to sex and age (+ 2 years). Descriptions of all demographic and clinical characteristics of cases and controls are given in Table 1.

**Table 1:** The baseline conditions of the participants

	Not Drepressed (*N* = 440)	Depressed (*N* = 110)
Age	68.00 (64.00–76.00)	68.00 (64.00–76.00)
Gender		
Male	168 (38.2%)	42 (38.2%)
Female	272 (61.8%)	68 (61.8%)
Kinds of hypoglycemic drugs		
No medication	90 (20.5%)	31 (28.2%)
Metformin	250 (56.8%)	45 (40.9%)
Other hypoglycemic drugs	100 (22.7%)	34 (30.9%)
Fasting blood-glucose		
< 7	175 (39.8%)	46 (41.8%)
7~11.1	159 (36.1%)	41 (37.3%)
> 11.1	106 (24.1%)	23 (20.9%)
HbA1c		
< 7	230 (52.3%)	68 (61.8%)
7~9	151 (34.3%)	32 (29.1%)
> 9	59 (13.4%)	10 (9.1%)
BMI		
< 24	164 (37.3%)	26 (23.6%)
24~28	208 (47.3%)	68 (61.8%)
≥ 28	68 (15.5%)	16 (14.5%)
Education		
Primary and below	90 (20.5%)	16 (14.5%)
Middle school	272 (61.8%)	66 (60.0%)
College and above	78 (17.7%)	28 (25.5%)
Companion		
Absent	357 (81.1%)	91 (82.7%)
Present	83 (18.9%)	19 (17.3%)
Pension		
Absent	407 (92.5%)	97 (88.2%)
Present	33 (7.5%)	13 (11.8%)
Sleep duration		
6~8	306 (69.5%)	67 (60.9%)
< 6	112 (25.5%)	39 (35.5%)
> 8	22 (5.0%)	4 (3.6%)
Physical ability and activity		
Good	282 (64.1%)	44 (40.0%)
Poor	158 (35.9%)	66 (60.0%)
Tobacco use		
Never	360 (81.8%)	88 (80.0%)
Current	55 (12.5%)	13 (11.8%)
Former	25 (5.7%)	9 (8.2%)
Alcohol use		
Never	397 (90.2%)	93 (84.5%)
Current	33 (7.5%)	15 (15.6%)
Former	10 (2.3%)	2 (1.8%)
Number of additional illnesses		
0	155 (35.2%)	26 (23.6%)
1~2	270 (61.4%)	66 (60.6%)
> 2	15 (3.4%)	18 (16.4%)

*Abbreviations*: *BMI* Body Mass Index. Age, *P* values are significant at α = 0.05

### 1:4 Matched univariate logistic regression analysis

Univariate logistic regression analysis showed that diabetic subjects taking metformin had a lower risk of depression than those taking no medication, with an adjusted OR of 0.506 and a 95% CI of 0.299–0.856 (*P* < 0.05). However, patients taking other hypoglycemic drugs showed no significant differences from controls with respect to the prevalence of depression. Those with depression were more likely to be overweight, have poorer physical capabilities and activity, or manifest more than two additional illnesses. All differences were statistically significant at *P* < 0.05. There were no significant differences in the prevalence of depression among groups with respect to fasting blood-glucose, HbA1c, education, presence of a companion, having a pension, sleep duration, alcohol use, or smoking status (*P* > 0.05) (Table 2).

**Table 2:** The results of univariate analysis on the influencing factors of depression in participants with diabetes mellitus

	OR	95.0% *CI* for OR		*P*
		Lower	Upper	
Kinds of hypoglycemic drugs	0.010			
No medication	Ref			
Metformin	0.506	0.299	0.856	0.011
Other hypoglycemic drugs	1.003	0.576	1.748	0.990
Fasting blood-glucose	0.768			
< 7	Ref			
7~11.1	0.978	0.615	1.556	0.925
> 11.1	0.817	0.463	1.443	0.486
HbA1c	0.170			
< 7	Ref			
7~9	0.718	0.449	1.147	0.166
> 9	0.563	0.269	1.177	0.127
BMI	0.015			
< 24	Ref			
24~28	2.106	1.269	3.493	0.004
≥ 28	1.484	0.743	2.965	0.263
Education	0.098			
Primary and below	Ref			
Middle school	1.430	0.762	2.685	0.265
College and above	2.156	1.051	4.425	0.036
Companion				
Absent	Ref			
Present	0.882	0.488	1.595	0.678
Pension				
Absent	Ref			
Present	1.648	0.836	3.246	0.149
Sleep duration	0.103			
6~8	Ref			
< 6	1.617	1.020	2.563	0.041
> 8	0.854	0.290	2.512	0.774
Physical ability and activity				
Good	Ref			
Poor	2.717	1.753	4.211	0.000
Tobacco use	0.610			
Never	Ref			
Current	1.036	0.506	2.121	0.923
Former	1.533	0.645	3.647	0.334
Alcohol use	0.120			
Never	Ref			
Current	2.022	1.025	3.991	0.042
Former	0.971	0.196	4.815	0.971
Number of additional illnesses	0.000			
0	Ref			
1~2	1.489	0.916	2.423	0.109
> 2	7.026	3.128	15.782	0.000

*P* values are significant at α = 0.05

*Abbreviations*: *CI* confidence interval, *BMI* Body Mass Index, *OR* Odds Ratio

### 1:4 Matched multivariate logistic regression analysis

The factors showing statistical differences in the above univariate analysis were then introduced into the multivariate paired logistic model for a stepwise regression analysis. The results showed that type of hypoglycemic drug, BMI, physical capability and activity, and number of additional illnesses were statistically significant; i.e., overweight status, poor physical capability and activity, and more than 2 additional illnesses were risk factors for depression in elderly patients with diabetes—with an adjusted OR of 2.031 and a 95% CI of 1.180–3.495, an adjusted OR of 2.342 and 95% CI of 1.465–3.743, and an adjusted OR of 5.350 and 95% CI of 2.222–12.883, respectively (*P* < 0.05 for all comparisons). Patients who took metformin had a lower risk of depression than those taking no medication, with an adjusted OR of 0.567 and 95% CI of 0.323–0.997 (*P* < 0.05), while patients taking other hypoglycemic drugs showed no significant differences from patients who did not take any medication (Table 3).

**Table 3:** The results of multivariate analysis of influencing factors of depression in participants with diabetes mellitus

	OR	95.0% *CI* for OR		*P*
		Lower	Upper	
Kinds of hypoglycemic drugs	0.049			
No medication	Ref			
Metformin	0.567	0.323	0.997	0.049
Other hypoglycemic drugs	1.063	0.592	1.907	0.839
BMI	0.023			
< 24	Ref			
24~28	2.031	1.180	3.495	0.011
≥ 28	1.161	0.553	2.436	0.694
Physical ability and activity				
Good	Ref			
Poor	2.342	1.465	3.743	0.000
Number of additional illnesses	0.001			
0	Ref			
1~2	1.378	0.832	2.284	0.213
> 2	5.350	2.222	12.883	0.000

Adjusted for kinds of hypoglycemic drugs, BMI, educational status, companion status, pension, sleep duration, physical ability and activity, tobacco use, alcohol use, number of additional illness. Only data with significantly difference was shown in the table

*P* values are significant at α = 0.05

*Abbreviations*: *CI* confidence interval, *BMI* Body Mass Index, *OR* Odds Ratio

## Discussion

To the best of our knowledge, this is the first matched case–control study to evaluate the risk factors for depression in elderly diabetic patients and also the first to assess the association of metformin with the depressive state.

Some investigators have established that age, female gender, low family income, lower educational levels, higher HbA1c levels, and higher BMI are risk factors for concomitant depression in individuals with T2DM [33, 34]. Such individuals feel dissatisfied with their lives, abandon many of their activities and interests, and feel an emptiness to their lives. However, diabetes control can be improved by increasing a patients’ self-efficacy and ability to care for their own illnesses [35]. Notably, female patients with diabetes are likely to have a higher prevalence of depressive symptoms than men. Another study reported that female gender, age, poor glycemic control, obesity, diabetic complications, and insulin therapy in the Chinese population were risk factors for T2DM when combined with depression [36]. However, there are also studies establishing that the association of diabetes and depression was independent of an individual’s education and household income. Additionally, the presence of depression was not affected by other sociodemographic factors, BMI, hypertension, or the number of diabetes-associated complications [37]. We here used a 1:4 matched case–control design, which was different from the cross-sectional research design reported in the aforementioned study. In the current study, the participants were over 60 old; and their age and gender were matched to those of the controls, since variability in these factors might have an untoward impact on the results. Case matching was performed to render the research more efficient and to ensure the reliability of the results. In the present study we suggest that the association of diabetes and depression in the elderly was independent of companionship, having a pension, and tobacco use. This may be because a high proportion (81.45%) of people in our group had partners and a high proportion (91.6%) had pensions. Smokers constituted only 12.4% of the total. Also, we reported that neither poor fasting blood glucose control nor high HbA1c levels increased the risk of depression in elderly diabetics. This may be because our research population was not a completely cross-sectional study group and that the number of cases was relatively small. Additionally, in our study, the relative score of our depressed population was lower and the degree of depression was milder. Among the 110 cases, 86 exhibited mild depression, which may have had an effect on the results.

Although there were no significant differences in the prevalence of depression among elderly diabetic patients with respect to sleep duration (overall *p* value > 0.05), our study showed that relatively longer sleep duration prone to protect diabetes patients from depressive symptoms, which was similar to previous studies [8, 38]. However, no consistent conclusion as to the effect of educational level on depression: some investigators have observed that people with a lower educational level are prone to depression [33, 34], while others observed that there was no correlation between educational level and depression [14, 37]. Our study also show that, in elderly patients with diabetes mellitus, there was no correlation between educational level and depression although it seems that patients with higher educational background tended to manifest depression. It may be that more highly educated and elderly diabetic patients in China tend to obsess over the disease and become more prone to depression. This was similar to a study in China where ruminative thinking was a predictor of future depressive symptoms among the elderly in nursing homes [39]. The effect of alcohol consumption on depression is also controversial, and our study showed that there was no correlation between alcohol use and depression, although the prevalence of depression in elderly diabetic with current alcohol use was higher than in non-drinkers.

Multivariate analysis revealed that, among elderly diabetic patients, those who were overweight, with poor physical capability and activity, and having multiple additional illnesses, had an increased risk of depression; while the use of metformin decreased the risk of depressive symptoms. We, however, reached no uniform conclusion as to the relationship between BMI and depression. While some studies have also shown a lack of correlation between BMI and depression [8, 40], other investigators observed that BMI was associated with depression [41, 42]. The risk of depression increased with increasing BMI, but the average BMI in our study was less than 24 [36]. Studies have also reported that changes in BMI were significantly closely correlated with changes in depression, i.e., that patients who lost more weight experienced greater improvements in depressive symptoms [43–45]. Our study showed that the prevalence of depression in the overweight group was higher than that in the normal-weight group (although this difference was not statistically significant, the incidence of depression tended to be higher with obesity). It is conceivable that Chinese are generally thinner than people from other countries. Once people become overweight, they may then become more depressed as their BMI increases; although obese people may not care as much about weight, which may leave them less anxious. Elderly people who have good physical capabilities and activity can generally go for walks, exercise, and talk with people in the community; and as a result their mood can be relatively pleasant, with a lower risk of depression. This is similar to the conclusions reported by Narita et al., where physical activity helped ameliorate depression in patients with diabetes [46]. Elderly people with multiple diseases are also likely to suffer from depression, which is similar to the findings of another recent study [47]. One reason for this may be the possibility of increased physical discomfort and increased psychologic burden caused by multiple diseases, and the another reason might be that the disease involves greater financial costs.

Metformin is a first-line hypoglycemic drug for type 2 diabetics. Recent research, however, has suggested that metformin also exerts an antidepressant effect through improvement of cognitive function in depressed patients with diabetes mellitus [22]. Additionally, it has been reported that both metformin monotherapy and its metformin used in combination with telmisartan can normalize depressive moods, reduce proinflammatory mediators, and ameliorate dysfunctions of the hypothalamic-pituitary-adrenal (HPA) axis, thereby providing beneficial effects in diabetes-induced depression [48]. In older men with type 2 diabetes, metformin reduced the likelihood of depression by 5.0, 2.8, and 15.6% in the high cancer risk class, the high CVD risk class, and the high frailty risk class, respectively [49]. Shivavedi observed that monotherapy with metformin and combination therapy with both metformin and ascorbic acid induced significant reductions in plasma corticosterone concentrations and adrenal weight. The effects of metformin and metformin in combination with ascorbic acid also caused significant reductions in oxidative stress and proinflammatory cytokines. Our current study suggests that metformin therapy could be a potential strategy to treat T2DM and co-morbid depression [10]. One study showed that individuals with undiagnosed depression might be at increased risk for non-adherence to metformin use [50], and other studies have shown that metformin does not improve depression. For example, for patients with post-stroke depression combined with T2DM, metformin did not ameliorate the depressive symptoms [51]. Co-administration of pioglitazone or metformin with low-dose fluoxetine did, however, improve mechanical allodynia, thermal hyperalgesia, and neurohistopathologic changes; and co-administration of pioglitazone improved depressive-like behavior in the peripheral nerve injury model of neuropathic pain in rats but co-administration of metformin, did not [52]. It was also reported that metformin had minimal effects on depressive symptoms (comparable to placebo), although it changed the HOMA-IR [53]. One underdiagnosed side effect of metformin is the increased risk of cobalamin (vitamin B12) deficiency due to diminished uptake of cobalamin by the terminal ileum, and cobalamin deficiency was associated with an increased risk of depression and decreased cognitive performance. It follows, then, that metformin might increase the risk of depression [54]. However, in our study, we found that in elderly diabetic patients, those on metformin exhibited a lower risk of depression than those on other hypoglycemic drugs or those on no medication—a finding similar to that reported in previous studies in which metformin alleviated depression [10, 22, 48, 49]. The mechanisms underlying this finding, however, require further study.

One advantage of this study is the close matching of cases to controls. Because the purpose of this work was to assess the relationship between metformin and depression, we controlled for the age and gender of the study participants at the recruitment stage, considering both potential confounding factors. Both cases and controls were recruited by the same research assistant from the same population of the same source to reduce confounding bias. The study also had a number of limitations. First, all health-related diseases except diabetes were self-reported, and because the study was a matched design, the associations of age and gender with depression in study participants could not be determined. Second, the subjects with diabetes in our study had all received treatment, and we did not include any participants who were newly diagnosed. One possible influencing factor for depression that was not taken into account was the duration of diabetes mellitus. Fourth, because many of the participants who had depression or other mental illnesses might have been taking anti-depressant medications or antipsychotics, and since these medications may reduce the burden of depressive symptoms and thus reduce the association of risk factors with current symptoms, we excluded them. Fifth, the group of metformin users included only those who used metformin or those who used metformin along with other hypoglycemic drugs. The control group was treated with monotherapy or a combination of hypoglycemic drugs other than metformin. We did not evaluate effects of hypoglycemic drugs other than metformin between the two groups. There were also some defects in our study design, but the observed association between metformin and depression was attributable to the effects of metformin alone and not the interactive effects of metformin with other hypoglycemic drugs. Such factors could conceivably create some study bias.

## Conclusions

In summary, we here showed that the risk factors for depression in elderly patients with diabetes mellitus are complicated, including overweight status, poor physical capabilities and activity, and more than two additional illnesses. While these were risk factors for depression in elderly diabetic patients, metformin was found to be a protective factor against depression in this group.

## Data Availability

Inquiries regarding the availability of primary data should be directed to the principal investigator, Professor Difei Wang.

## Abbreviations

BMI: Body mass index
CIs: Confidence intervals
FPG: Fasting plasma glucose
GDS-15: Geriatric Depression Scale-15
HPA: Hypothalamic-pituitary-adrenal axis
ORs: Odds ratios
T2DM: Type 2 diabetes mellitus
UN: United Nations
WC: Waist circumference
